# Proposal of e-learning strategy to teach Atraumatic Restorative Treatment (ART) to undergraduate and graduate students

**DOI:** 10.1186/1756-0500-7-456

**Published:** 2014-07-17

**Authors:** Lucila Basto Camargo, Daniela Prócida Raggio, Carlos Felipe Bonacina, Chao Lung Wen, Fausto Medeiros Mendes, Marcelo José Strazzeri Bönecker, Ana Estela Haddad

**Affiliations:** 1 Discipline of Pediatric Dentistry, Faculdade de Odontologia da Universidade Paulista, Av. Comendador Enzo Ferrari, 280 - Swift, Campinas, Brazil; 2 Department of Pediatric Dentistry, Faculdade de Odontologia da Universidade de São Paulo, Av. Professor Lineu Prestes, 2227, Cidade Universitária, São Paulo, Brazil; 3 Pediatric Dentistry Clinical Practioner São Paulo, São Paulo, Brazil; 4 Department of Pathology, Faculdade de Medicina da Universidade de São Paulo, Av. Dr. Arnaldo, 455 - Cerqueira César, São Paulo, Brazil

**Keywords:** E-learning, Dental education, Atraumatic Restorative Treatment, Caries

## Abstract

**Background:**

The aim of this study was to evaluate e-learning strategy in teaching Atraumatic Restorative Treatment (ART) to undergraduate and graduate students. The sample comprised 76 participants—38 dental students and 38 pediatric dentistry students—in a specialization course. To evaluate knowledge improvement, participants were subjected to a test performed before and after the course.

**Results:**

A single researcher corrected the tests and intraexaminer reproducibility was calculated (CCI = 0.991; 95% IC = 0.975–0.996). All students improved their performances after the e-learning course (Paired t-tests p < 0.001). The means of undergraduate students were 4.7 (initial) and 6.4 (final) and those of graduate students were 6.8 (initial) and 8.2 (final). The comparison of the final evaluation means showed a statistically significant difference (t-tests p < 0.0001).

**Conclusions:**

The e-learning strategy has the potential of improving students’ knowledge in ART. Mature students perform better in this teaching modality when it is applied exclusively via distance learning.

## Background

Dental caries prevalence has declined in recent years [1] but still causes negative impact on patients’ quality of life [2]. Currently, undergraduate dental education has been widely discussed in many countries with the aim of modernizing the curriculum in cariology. Undergraduate dental education should enable students to become professionals with critical potential who are able to perform early diagnosis and have a wider understanding of the caries process according to the best scientific evidence available [3,4].

New options for preventive measures, as well as operative and non-operative treatments, have been described and should be taught as they present scientific evidence of effectiveness [5-8]. Among these options, we can emphasize Atraumatic Restorative Treatment (ART), which was advocated in the early ‘80s and was officially adopted by the World Health Organization in the ‘90s [9]. ART is a definitive restorative treatment that is low cost and is based on minimal intervention involving prevention, early interception and tooth structure preservation [10].

An interesting strategy to face the current challenges of contemporary education is the inclusion of e-learning in the undergraduate curriculum [11,12]. It enables the merger of face-to-face learning activities with online learning experiences that allow student-centered learning and efficient use of time [13]. It is effective and presents results that are similar to [14,15] or even better than the traditional methodology [16]. Some studies also showed that e-learning can enhance the learning experience when it is used as a support to presentational teaching [17-19]. Besides that, it is well received by dental students, which makes it desirable for undergraduate courses [20-22].

In a previous study [23], we evaluated the benefits of an e-learning training course on ART that was applied to practicing dentists and found encouraging results. In this study, we applied this same course to undergraduate and graduate dental students to evaluate the potential of an e-learning strategy in teaching ART.

## Methods

The Local Ethics Committee in Research (Ethics Committee in Research –UNIP – Paulista University) approved this study (protocol 97.085 – 09/08/2012) and the participants received written information and signed a consent form.

The Departments of Pediatric Dentistry and Telemedicine – University of São Paulo elaborated a DVD training course in ART (about 40 minutes). This e-learning course combined many resources, such as the “Virtual Man project,” clinical videos, interviews with ART experts, clinical pictures and radiographs. Its elaboration was better described in the previous study [23].

**Figure 1:**
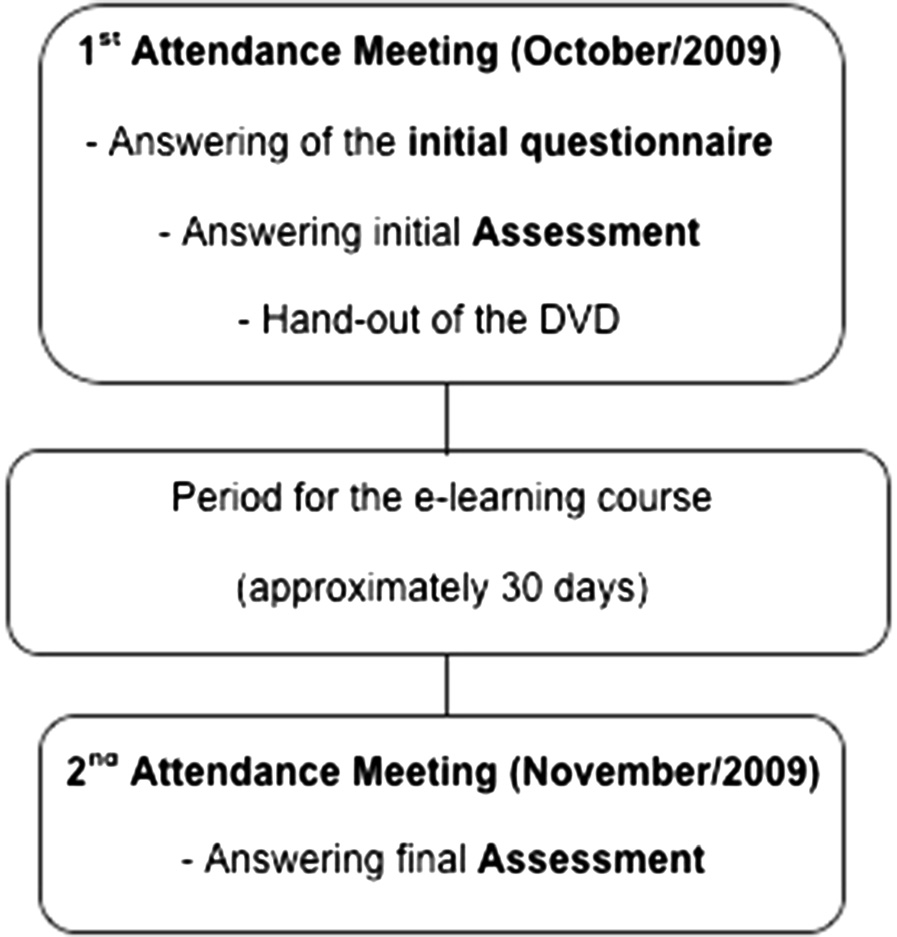
Flow chart of assessment activities.

The sample consisted of undergraduate dental students (5th and 7th semesters from a total of 8) (undergraduate group) and pediatric dentistry students (graduate group). The inclusion criteria had no restrictions regarding sex or age.The students answered a multiple-choice questionnaire at baseline. This questionnaire gathered information on the dentists’ initial knowledge of ART, their beliefs on the treatment technique and their interest in the course. They received the DVD and had approximately 30 days to assess the e-learning course. To evaluate knowledge improvement (competence level), participants were subjected to a test performed before (Assessment 1) and after (Assessment 2) the course (Figure 1).

The test consisted of multiple-choice and open-ended questions in three sections. Section 1 comprised questions on theoretical/conceptual/cognitive skills, with fifty sentences that could be true or false, addressing various topics of ART. Section 2 had written tests that comprised cognitive reasoning-contextualization, with five clinical situations illustrated with photographs and radiographs. Section 3 also had a written test regarding observation in the clinical setting, with five short films from clinical videos and/or the Virtual Man project. The film sections were exhibited twice for all participants at the same time, with an interval of thirty seconds between each exhibition. The three sections, combined, assessed the dentists’ theoretic-scientific knowledge on the topic, evaluated their ability to make decisions in different circumstances, and tested their adeptness in identifying aspects of different ART-related situations and procedures. The maximum time allowed for each section was thirty-five minutes.

In a previous study [23], due to the subjectivity involved when correcting the written answers, two researchers underwent a calibration exercise. One of these researchers performed the correction of all tests in the present study (intraexaminer reproducibility CCI = 0.991; 95% IC = 0.975–0.996).

To evaluate the performance of each student, a paired t-test was used to compare the means between grades from Assessment 1 (before course) and 2 (after course). To compare the groups’ performance (undergraduate and graduate), we applied the Student’s t-test to the Assessment 2 mean (after the course),

## Results

One hundred and twenty undergraduate students were invited to participate in the study. Thirty-eight of these performed the evaluation process completely; and, of 41 graduate students invited, 38 finished the survey (Response rate: undergraduate group 38%; graduate group, 93%). Thus, the e-learning strategy was evaluated through the performance of 76 students who attended the course.

Data from the initial questionnaire are summarized in Tables 1 and 2.

**Table 1:** Personal data from the undergraduate and graduate groups

		**Undergraduate**	**Graduate**
**Age (years)**	Median Range	(19–44)	(22–42)
	19–24 years	20 (52.6%)	14 (36.8%)
	25–44 years	18 (47.4%)	24 (63.2%)
**Sex**	Male	9 (23.7%)	1 (2.6%)
	Female	29 (76.3%)	37 (97.4%)

**Table 2:** Students’ background in ART

	**Undergraduate**	**Total**	**Graduate**	**Total**
Have used ART before	7 (18.4)	38 (100)	34 (89.5)	38 (100)
Never used ART	31 (81.5)		4 (1.5)	
Lack of training	30 (78.9)	38 (100)	13 (34.2)	38 (100)
Lack of restorative material	1 (2.6)		24 (63.2)	
Negative previous experience	0 (0)		0 (0)	
Did not answer	7 (18.4)		1 (2.6)	
Defends ART	29 (76.3)	38 (100)	36 (94.7)	38 (100)
Does not defend ART	6 (15.7)		2 (5.3)	
Did not answer	3 (7.8)		0 (0)	
Use only as urgent/temporary treatment	18 (47.36)	38 (100)	12 (31.6)	38 (100)
Use as definitive treatment	17 (44.73)		26 (68.4)	
Did not answer	2 (5.26)		0 (0)	

The mean grades of each student before and after the course were compared, initially considering each sector of the test and also the means of these three sectors, showing a statistically significant difference (p <0.001). Students from both groups showed ART knowledge improvement at all levels of comparison performed.

All grades achieved in the evaluation process are presented in Table 3.

**Table 3:** Students’ grades from the evaluation process

	**Undergraduate**			**Graduate**		
	**Initial**	**Final**	P value*	**Initial**	**Final**	P value*
Section 1	6.2	8.2	<0.0001	8.3	9.4	<0.0001
Section 2	3.7	5.0	<0.0002	5.8	7.1	<0.0001
Section 3	4.2	6.0	<0.0001	6.3	8.1	<0.0001
Average of the 3 sections	4.7	6.4	<0.0001	6.8	8.2	<0.0001

*Student’s t-test.

The comparison of the final evaluation grades between the two groups showed a statistically significant difference (p < 0.0001), indicating that graduate students finished the course with better performance than undergraduate students.

## Discussion

It is clear that dental students need systematic and consistent education in cariology [24]; e-learning can contribute to this process by providing advantages such as ample autonomy regarding where, how and when the student will dedicate himself to the educational process [18,25,26]. However, self-motivation, technical problems and lack of interaction between teachers and students when content is offered exclusively at distance are difficulties with the potential to cause negative impact upon the students’ performances [23,25]. Therefore, our goal was to evaluate the e-learning strategy in teaching ART for undergraduate and graduate students.

One finding of this study was that half of the undergraduate students, at baseline, showed an inadequate concept of ART and believed that it was a temporary and/or emergent treatment. This points out the need to properly teach ART in undergraduate courses [27], as the construction and consolidation of misconceptions with the student are more difficult to correct later on [23].

Our results demonstrate that a significant improvement of the students’ knowledge occurred after using the e-learning strategy. This improvement shows that a well-structured e-learning course can be a good alternative in teaching ART and helping implement scientific evidence-based treatments in clinical practice. Besides, graduate students achieved higher grades in the final evaluation (8,2) when compared to undergraduate students (6,4). We hypothesize that there is a relationship between students’ performance and their motivation in a distance-learning course, as observed previously [28].

It is noteworthy that the same learning strategies were able to sensitize and motivate the graduate students, but were not as effective with the undergraduate students. Also, the lack of using ART in clinical practice during their undergraduate courses may possibly have contributed to the poor performance presented by this group; conversely, the graduate group was composed of pediatric dentistry students, and one of the major indications of ART is its use in children to decrease anxiety levels [29].

Thus, in order to improve the undergraduate students’ performance, other teaching strategies should be linked to the learning process. Interaction with tutors should provide motivation, guidance and support to these students. This practice integration characterizes blended learning [30].

## Conclusions

In conclusion, the e-learning strategy has the potential of improving students’ knowledge of ART. Mature students perform better with this teaching modality when applied exclusively via distance learning.

## Competing interests

The authors declare that they have no competing interests.

## Authors’ contributions

LBC performed the experiment and wrote the manuscript. DPR experimental design, contributed substantially to the discussion and proofread the manuscript. CFB performed the experiment. CLW developed the idea and proofread the manuscript. FMM contributed substantially to the discussion and performed the statistical evaluation. MJSB proofread the manuscript and contributed substantially to the discussion. AEH developed the experimental design, contributed substantially to the discussion and proofread the manuscript. All authors read and approved the final manuscript.
